# Conversion of mild cognitive impairment patients in Alzheimer’s disease: prognostic value of Alpha3/Alpha2 electroencephalographic rhythms power ratio

**DOI:** 10.1186/s13195-015-0162-x

**Published:** 2015-12-29

**Authors:** D. V. Moretti

**Affiliations:** Alzheimer’ Disease Rehabilitation Unit, IRCCS S. Giovanni di Dio Fatebenefratelli, Brescia, Italy

## Abstract

**Introduction:**

The increase in electroencephalogram (EEG) alpha3/alpha2 frequency power ratio has been demonstrated as a biomarker characteristic of subjects with mild cognitive impairment (MCI) who will develop Alzheimer’s disease (AD).

**Methods:**

Seventy-four adult subjects with MCI underwent clinical and neuropsychological evaluation, EEG recording, and high-resolution 3D magnetic resonance imaging (MRI). This group has been evaluated after a three years follow-up. Twenty-seven of these subjects underwent perfusion single-photon emission computed tomography (SPECT) evaluation also. Increasing alpha3/alpha2 power ratio, was computed for each subject. Differences in EEG markers, cortical thickness, brain perfusion among the groups were estimated.

**Results:**

In the higher alpha3/alpha2 frequency power ratio group, greater memory impairment was correlated with greater cortical atrophy and lower perfusional rate in the temporo-parietal cortex. After a follow-up of three years, these patients converted in AD.

**Conclusion:**

High EEG upper/low alpha power ratio was associated with cortical thinning and lower perfusion in the temporo-parietal lobe. Moreover, atrophy and lower perfusion rate were both significantly correlated with memory impairment in MCI subjects. The increase of EEG upper/low alpha frequency power ratio could be useful for identifying individuals at risk for progression to AD dementia and may be of value in the clinical context.

## Introduction

Mild cognitive impairment (MCI) commonly represents the at-risk state of developing dementia. Therefore, there is a need to develop early biomarkers that allow identification of subjects who could develop the disease and would be useful for early diagnosis and effective prevention therapies. The identification and validation of biomarkers for diagnosing, monitoring progression, and predicting the onset of Alzheimer’s disease (AD) have been a main focus of AD research in the past ten years. In line with recently published research criteria, it is becoming clear that the integration of different biomarkers is a milestone for a correct and early diagnosis of AD [1]. To date, the most studied and validated biomarkers are Abeta42 and tau protein in the cerebrospinal fluid (CSF), glucose hypometabolism measured by fluorodeoxyglucose positron emission tomography (18F-FDG PET), atrophy of hippocampal volume (HV) on magnetic resonance imaging (MRI), and brain amyloid deposition measured by amyloid imaging with PET [2]. Recent MRI studies have demonstrated that a large neural network is altered in subjects with prodromal AD [3]. In particular, subjects with cognitive decline show early atrophy and loss of gray matter in cortical-specific brain areas including precuneus, hippocampal, medial temporal, and parietal lobes [4, 5]. In the conceptual frame of the integration of biomarkers for an early and highly predictive diagnosis, the electroencephalogram (EEG) could be a reliable tool [6]. Indeed, it is widely accepted that the cerebral EEG rhythms reflect the underlying brain network activity [7]. Therefore, modifications in EEG rhythms could be an early sign of AD. In particular, the study of alpha rhythm seems to be a very suitable tool to detect the relationship between structural and functional brain networks [8]. Previous studies have convincingly demonstrated that there are thalamo-cortical and cortico-cortical components that interact in the generation of cortical alpha rhythms [6]. Recently, it has been demonstrated that the increase of high alpha relative to low alpha power is both a reliable EEG marker of hippocampal atrophy [9] and predictive of conversion of patients with MCI to AD, but not to non-AD dementia [10]. On the other hand, subjects with higher alpha3/alpha2 frequency power ratios showed a constant trend toward a lower perfusion than the low alpha3/alpha2 group associated with an increase of theta frequency power [11, 12].

In this study, the correlation between MRI, single-photon emission computed tomography (SPECT), and memory impairment in the MCI group with higher alpha3/alpha2 frequency power ratio was investigated in order to detect a prognostic value of this EEG biomarker.

## Methods

### Subjects

For the present study, 74 subjects with MCI were selected from a prospective study on the natural history of cognitive impairment (the translational outpatient memory clinic—TOMC study) carried out in the outpatient facility of the National Institute for the Research and Care of Alzheimer’s Disease (IRCCS Istituto Centro San Giovanni di Dio Fatebenefratelli, Brescia, Italy). Patients with MCI underwent clinical and cognitive assessment and high-resolution MRI. A detailed description has been provided elsewhere [12]. The study protocol was approved by the local ethics committee, and all participants or their caregivers signed an informed participation consent according to the Code of Ethics of the World Medical Association (Declaration of Helsinki). The ethics committees involved were from: 1) S. John of God Hospital, Brescia (main committee); 2) Citta’ di Brescia’ Hospital, Brescia; and 3) Ospedali Riuniti Hospital, Bergamo.

### Diagnostic criteria

Patients were rated with a series of standardized diagnostic and severity instruments, including the Mini-Mental State Examination (MMSE) [13], the Clinical Dementia Rating Scale (CDRS) [14], the Hachinski Ischemic Scale (HIS) [15], and the Instrumental and Basic Activities of Daily Living (IADL, BADL) [16]. Subjects also underwent a complete neuropsychological battery assessing: memory (Babcock Story Recall – Rey–Osterrieth Complex Figure, Recall – Auditory-Verbal Learning Test, and immediate and delayed recall), verbal and non-verbal memory, attention, and executive functions (Trail Making Test B, A and B-A and Inverted Motor Learning-Clock Drawing Test;), abstract reasoning thinking (Raven Colored Progressive Matrices), frontal functions (Inverted Motor Learning), language (Phonological and Semantic fluency-Token test), and apraxia and visuo-constructional abilities (Rey–Osterrieth Complex Figure, Rey figure copy, and Clock Drawing Test) [17]. All the neuropsychological tests were standardized on an Italian population, thus scores were compared to normative values with age, education, and gender corrections in an Italian population [18, 19].

In addition, patients underwent diagnostic neuroimaging procedures [magnetic resonance imaging (MRI)] and laboratory testing to rule out other causes of cognitive impairment. MCI was defined as the presence of objective impairment in memory or other cognitive domains (performance lower than the fifth percentile on neuropsychological tests as detailed below) in the absence of functional impairment. History or neurological signs of major stroke, history of depression (from mild to moderate or major depression) or juvenile-onset psychosis, other psychiatric diseases, overt dementia, epilepsy, drug addiction, or alcohol dependence, use of psychoactive drugs, including acetylcholinesterase inhibitors or other drugs enhancing brain cognitive functions or biasing EEG activity, and current or previous uncontrolled or complicated systemic diseases (including diabetes mellitus) or traumatic brain injuries were absent. All subjects were right-handed. These inclusion and exclusion criteria for MCI were based on previous seminal studies [1]. As the aim of our study was to evaluate the relationship between gray matter (GM**)** loss and alpha2/alpha3 ratios in MCI subjects, we did not consider the clinical subtype of MCI, i.e., amnesic or non-amnesic, single or multiple domains. Demographic and cognitive features of the subjects in this study are summarized in Table 1. There were no statistically significant differences in age, gender, or education among the groups in this study.

**Table 1:** Demographic and cognitive characteristics of the whole sample, disaggregated for increased levels of alpha3/alpha2

Alpha3/Alpha 2 power ratio					
Demographic and clinical future	High	Middle	Low	p	F
Number of subjects	18	38	18	---	---
Age years	70.4 ± 6.7 [60-85]	68.4 ± 8.2 [52-83]	70.4 ± 7.4 [57-80]	.55	0,6
Sex, female	13 (%)	24 (%)	14 (%)	.51	0,67
Education, years	6.6 ± 3.6 [4–18]	7.6 ± 3.7 [3–17]	8.3 ± 4.7 [3–18]	.42	0,89
Mini Mental State Exam	27 ± 1.7 [23–29]	27.4 ± 1.3 [24–30]	26.9 ± 1.2 [23–30]	.46	2,46
Alpha3/alpha2	1.29 ± 0.1 [1.17-1.52]	1.08 ± 0.0 [1-1.16]	0.9 ± 0.1 [0.77-0.98]	.000	144,62

Numbers denote mean ± standard deviation, number and [range]. p denotes significance on analysis of variance (ANOVA)

### Follow-up analysis

Table 2 shows the socio-demographical features of three groups of patients that were obtained according to clinical outcome after three years of follow-up: 1) MCI who did not convert to any dementia; 2) MCI who converted to AD; and 3) MCI who converted to dementia other than AD [fronto-temporal spectrum (FTD), vascular dementia (VD), or Lewy body dementia,(LBD)].

**Table 2:** ANOVA results of demographic variables, i.e., age, education, MMSE score, and alpha3/alpha2 ratio (see text for details)

Demographic and clinical features	MCI cohort	MCI NON CONVERTERS	MCI AD CONVERTERS	MCI NON-AD CONVERTERS	p value (ANOVA)
Number of subjects (f/m)	74 (46 F/28 M)	42 (30/14)	18 (10/8)	14 (7/7)	
Age (years)	69.7 ± 2.3	69.4 ± 4.1	71.8 ± 5.7	70.1 ± 3.9	0.1
Education (years)	7.4 ± 0.8	7.3 ± 2.5	8.4 ± 3.1	8.8 ± 4.6	0.4
MMSE	26.8 ± 1.4	27.2 ± 1.4	25.7 ± 1.5	26.8 ± 1.8	0.001
alpha3/alpha2 ratio	1.15 ± 0.2	1.14 ± 0.3	1.26 ± 0.5	1.03 ± 0.7	0.02

The values are all intended at baseline. *ANOVA* analysis of variance, *MMSE* Mini-Mental State Examination, *MCI* mild cognitive impairment, *AD* Alzheimer’s Disease

### EEG recordings

The EEG activity was recorded continuously from 19 sites by using electrodes set in an elastic cap (Electro-Cap International, Inc. Eaton, OH, USA) and positioned according to the 10–20 international system. (Fp1, Fp2, F7, F3, Fz, F4, F8, T3, C3, Cz, C4, T4, T5, P3, Pz, P4, T6, O1, and O2). The patients were instructed to sit with closed eyes and relax, constantly monitored by an operator. The ground electrode was placed in front of Fz. The left and right mastoids served as the reference points for all electrodes*.*

The recordings were used off-line to re-reference the scalp recordings to the common average. Re-referencing was done prior to the EEG artifact detection and analysis. A simple bimastoid referential derivation could have the effect of distorting the EEG signal, introducing spurious, non cerebral, activity. Data were recorded with a band-pass filter of 0.3–70 Hz and digitized at a sampling rate of 250 Hz (BrainAmp, BrainProducts, Munich, Germany). Electrode-skin impedance was set below 5 kilo-ohms. Horizontal and vertical eye movements were detected by electrooculogram (EOG). The recording lasted 5 min with the subjects eyes closed. EEG data were then analyzed and fragmented off-line in consecutive epochs of 2 s with a frequency resolution of 0.5 Hz. The average number of epochs analyzed was 140, ranging from 130 to 150. The epochs with ocular, muscular, and other types of artifacts were discarded by two skilled electroencephalographists. The spectral power we obtained is an estimation of a spectrum collapsed all over the scalp electrodes. In this way, the eventual contribution of the muscular artifact is strongly reduced. The spectral power was averaged across all electrodes to obtain a sort of global field power, which would have reduced the channel to channel variability, with the advantage of extracting a high stationary measure and of obtaining a smoother, clearer, and homogeneous individual alpha peak [10]. Moreover, this method has been chosen because it is not affected by the low spatial resolution of EEG. It should be possible to compute a more focused field, choosing a subset of electrodes next to the brain region of interest. However, this procedure has two main disadvantages: 1) it needs a larger array of electrodes, given the volume conduction phenomenon. Of note, a larger electrode array should require the application of some further computation such as, for example, Laplacian filter or spline interpolation; this aspect is of particular importance because it is more time-consuming and not applicable in a clinical context; and 2) the power computation on a smaller number of electrodes could give rise to artefactual detection of the individual alpha frequency peak as the presence of double peak or the absence of a clear peak. These computation errors are overlooked by the power spectra computation collapsed on the whole array of electrodes. For the same reason, i.e., the lack of information about EEG cortical generators with conventional EEG, the correlation of structural or metabolic data with a focal (or single) electrode array, would give rise to poor information about the brain oscillations in that point. Finally, the reason for the choice of a global field power is the continuous interplay inside the brain of different oscillation networks, so that it is an integrated activity more than split focal loops. The reliability of the relationship between a global field potential and the underlying cortical activity has been recently demonstrated by Brunet and colleagues [20]. On the other hand, it should be advisable that further studies confirm these results obtaining a more detailed spatial resolution with some other methods, such as LORETA (low resolution electromagnetic tomography). With the aim of verifying the reliability of our collapsed method, we evaluated the correlation of the total alpha frequency band of global field power with the same alpha frequency in two macroareas, the frontotemporal and the parietoccipital regions. The Pearson’s correlation resulted in positive high correlations of, respectively 0.85 (p < 0,005) and 0.91 (p < 0,001).

### Analysis of individual frequency bands

All recordings were obtained in the morning with subjects resting comfortably. Vigilance was continuously monitored in order to avoid drowsiness. A digital FFT-based power spectrum analysis (Welch technique, Hanning windowing function, no phase shift) computed the power density of EEG rhythms (ranging from 2 to 45 Hz) with a 0.5 Hz frequency resolution. Two anchor frequencies [the theta/alpha transition frequency (TF) and the individual alpha frequency (IAF) peak] were selected according to the literature guidelines [21]. IAF and TF were computed for each subject in the study. These anchor frequencies were computed on the power spectra averaged across all recording electrodes. This “collapsed spectrum method,” being a normalized scalp spectrum, allows for the identification of a robust and reliable IAF. As a consequence, the computation of an individual alpha frequency peak was mandatory for our study. The TF marks the transition frequency between the theta and alpha bands and represents an estimate of the frequency at which the theta and alpha spectra intersect. TF was computed as the minimum power in the alpha frequency range since our EEG recordings were performed at rest. The IAF represents the frequency with the maximum power peak within the extended alpha range (5–14 Hz). Based on TF and IAF, we estimated the frequency band range for each subject as follows: delta from TF-4 to TF- 2, theta from TF-2 to TF, low alpha band (alpha1 and alpha2) from TF to IAF, and high alpha band (or alpha3) from IAF to IAF + 2. The alpha1 and alpha2 bands were computed for each subject as follows: alpha1 from TF to the midpoint of the TF-IAF range, and alpha2 from this midpoint to the IAF peak. The mean frequency range computed in MCI subjects considered as a whole are: delta 2.9–4.9 Hz; theta 4.9–6.9 Hz; alpha1 6.9–8.9 Hz; alpha2 8.9–10.9 Hz; alpha3 10.9–12.9 Hz. Finally, in the frequency bands determined on an individual basis, we computed the relative power spectra for each subject. The relative power density for each frequency band was computed as the ratio between the absolute power and the mean power spectra from 2 to 45 Hz. The alpha3/alpha2 was computed in all subjects and three groups were obtained according to increasing tertile values of alpha3/alpha2: low (a3/a2 < 1;) middle (1 < a3/a2 < 1.16) and high (a3/a2 > 1.17). The tertile division allows a balanced distribution of the study samples with the advantage of avoiding the extreme value in the statistical analysis. The three groups of MCI have been shown in previous studies to be different in nature. In particular, the high alpha3/alpha2 EEG power ratio MCI group is at major risk to convert to Alzheimer’s disease [11] compared to the other alpha3/alpha2 power ratio MCI groups. Moreover, we utilized this group subdivision for homogeneity and comparability with the previous studies.

### MRI scans

For each subject, a high-resolution sagittal T1 weighted volumetric MR scan was acquired in the Neuroradiology Unit of the ‘Citta’ di Brescia’ Hospital, Brescia, by using a 1.0 T Philips Gyroscan scanner with a gradient echo 3D technique: TR = 20 ms, TE = 5 ms, flip angle = 30, field of view = 220 mm, acquisition matrix 256 x 256 mm, slice thickness 1.3 mm.

### Cortical thickness estimation steps

Cortical thickness measurements for 74 MCI patients were made using a fully automated magnetic resonance imaging-based analysis technique: FreeSurfer v5.1.0, a set of software tools for the study of cortical and subcortical anatomy. Briefly, in the cortical surface stream, the models of the boundary between white matter and cortical gray matter, as well as the pial surface, were constructed. Once these surfaces are known, an array of anatomical measures becomes possible, including cortical thickness, surface area, curvature, and the surface normal at each point on the cortex [22].

### Single subject analysis

For each subject, the T1-weighted, anatomical 3-D MRI dataset was converted from Dicom format into .mgz format, intensity variations were corrected, and a normalized intensity image was created. The volume was registered with the Talairach atlas through an affine registration. Next, the skull was stripped using a deformable template model [22], and extracerebral voxels were removed. The intensity was normalized, and the skull-stripped image was then operated on by a segmentation procedure based on the geometric structure of the gray–white interface. Voxels were classified as white or gray matter, and cutting planes were selected to separate the hemispheres from each other. A white matter surface was then generated for each hemisphere by tiling the outside of the white matter mass for that hemisphere. Cortical thickness measurements were obtained by calculating the distance between those surfaces (white and pial surfaces) at each of approximately 160,000 points per hemisphere across the cortical mantle [22].

### Group analysis

In order to relate and compare anatomical features across subjects, it was necessary to establish a mapping that specifies a unique correspondence between each location in one brain and the corresponding location in another. Thus, the pial surface of an individual subject was inflated to determine the large-scale folding patterns of the cortex and subsequently transformed into a sphere to minimize metric distortion. The folding patterns of the individual were then aligned with an average folding pattern using a high-resolution surface-based averaging. Thickness measures were mapped to the inflated surface of each participant’s brain reconstruction, allowing visualization of data across the entire cortical surface. Finally, cortical thickness was smoothed with a 20-mm full-width at half-height Gaussian kernel to reduce local variations in the measurements for further analysis [22].

### Statistical analysis

Differences between groups in sociodemographic and neuropsychological features were analyzed using SPSS version 13.0 (SPSS, Chicago, IL, USA) performing an analysis of variance (ANOVA) for continuous variables and paired χ^2^ test for dichotomous variables. For continuous variables, post-hoc pairwise comparisons among groups were performed with the Games-Howell or Bonferroni tests, depending on the homogeneity of variance tested with Levene’s test.

Concerning the neuroimaging analysis, the Qdec interface in Freesurfer software was used. A vertex-by-vertex analysis was carried out performing a general linear model to analyze whether any difference in mean cortical thickness existed between groups (low: a3/a2 < 1 μV^2^; middle: 1 < a3/a2 < 1.16 μV^2^; high: a3/a2 > 1.17 μV^2^). The following comparisons were carried out: High vs. Low, High vs. Middle, and Middle vs. Low. Age, sex, education, global cognitive level (MMSE score), and WMHs were introduced as covariates in the analysis to avoid confounding factors. We first tried to apply an appropriate Bonferroni multiple-comparison correction in our analysis (at p < 0.05 corrected). Unfortunately, no p-value survived after this correction. Thus, we choose to set a more restrictive significance threshold (than p < 0.05 corrected) at p < 0.001 uncorrected for multiple comparisons. Moreover, we considered as significant only the clusters that also were equal to or larger than 30 mm^2^. Finally, a surface map was generated to display the results on an average brain. For illustrative purposes, significance was set to a P-value of ≤0.01 uncorrected for multiple comparisons.

As a control analysis, in order to exclude casual relationships between EEG markers and cortical volumes, a correlation between brain areas and memory performance was examined. The correlation analysis was performed on the three samples separately (High a3/a2, Low a3/a2, Middle a3/a2) and the entire sample (High, Low, and Middle grouped together). An exploratory analysis of non-linear correlation did not fit into the purpose of testing our a priori hypothesis. Indeed, we choose to apply a measure of linear dependence led by our a priori hypotheses for which the MCI group with the greater cortical thinning and higher a3/a2 EEG level (indicating an incipient AD) should show a clear correlation with the memory tests performance, in the sense that an increase in cortical thinning corresponds to a decrease in memory performance, and vice versa. Indeed, even if in the cognitive tests scores there are no significant differences, we hypothesized that the MCI group with the greater cortical thinning and higher a3/a2 EEG level, indicating an incipient AD, should show a clear correlation with the memory tests performance. The correlation analysis on a vertex-by-vertex basis was performed specifically for the following neuropsychological memory test results: Babcock Test, Rey auditory verbal learning test (AVLT) immediate recall, and Rey AVLT delayed recall. The analysis was thresholded at p < 0.001 uncorrected for multiple comparisons while results were mapped at P-value of <0.005 uncorrected for illustrative purposes. Only the clusters that survived at the statistical threshold and were equal to or larger than 15 mm^2^ were considered as significant.

### Statistical follow-up analysis

Preliminarily, one-way ANOVAs were performed in order to verify that the alpha3/alpha2 relative power ratio was significantly different among groups.

Finally, a discriminant factor analysis was performed to verify if the same significant variables could be useful in discriminating subjects and could have a diagnostic value. The model was built as follows: variable selection was done with a stepwise method; tolerance was set to 0.01 and F to enter to 1,0.

### SPECT scan

Twenty-seven patients and 17 normal controls underwent a SPECT scan in the nuclear medicine department of the Ospedali Riuniti Hospital, Bergamo. Each subject received an intravenous injection of 925 MBq of technetium-99 m ethyl cysteinate dimer (^99m^Tc-ECD) in resting conditions, lying supine with eyes closed in a quiet, dimly lit room. Forty to sixty minutes after injection, brain SPECT was performed using a dual-head rotating gamma camera (GE Elscint Helix, Wauwatosa, WI, USA) equipped with low energy-high resolution, parallel hole collimators. A 128 × 128 pixel matrix, zoom = 1.5, was used for image acquisition with 120 views over a 360° orbit (in 3° steps) with a pixel size and slice thickness of 2.94 mm. Butterworth filtered-back projection (order = 7, cutoff = 0.45 cycles/cm) was used for image reconstruction and attenuation correction was performed using Chang’s method (attenuation coefficient = 0.11/cm). Images were exported in DICOM format.

### SPECT processing protocol

To achieve a precise normalization, we generated a study-specific SPECT template using both SPECT and MR scans of all patients and normal controls under study following a procedure described in detail elsewhere [21]. Briefly, we created a customized high-definition MR template, we converted SPECT scans to Analyze format using MRIcro (Columbia, SC, USA), and we coregistered them to their respective MR scans with SPM2 (SPM, Statistical Parametric Mapping, version 2; Functional Imaging Laboratory, London, UK). We normalized each MR to the customized MR template through a nonlinear transformation (cutoff 25 mm) and applied the normalization parameters to the coregistered SPECT. We obtained the customized SPECT template as the mean of all the latter normalized SPECT images. The creation of a study-specific template allows for better normalization since it accounts for low uptake in ventricular structures and cortical hypoperfusion effects frequently present in elderly patients. The following regions of interest (ROI) were chosen for perfusion analyses in each hemisphere from the Pick atlas by a sub-routine implemented on SPM2: frontal, parietal and temporal lobes, the thalamus, and the hippocampal-amygdalar complex [23].

### SPECT statistical analysis

All statistical analyzes were performed using SPSS software ver. 13.0. We investigated the significance of the difference between the two groups (MCI at low and at high risk to develop AD) in socio-demographic, clinical, and cognitive features using the χ^2^ test for categorical variables (sex and ApoE carriers) and Student’s independent t test for continuous variables (volumetric, perfusion features, and EEG frequencies). In all cases, we set the significance threshold at p < 0.05. Since native SPECT scans were coregistered to their respective MR images, and the study-specific SPECT template was coregistered to the high-definition MR template, all the normalized SPECT and MR images used for the statistical analysis were coregistered to the SPM standard anatomical space. Pearson’s r correlations were assessed between the selected perfusion ROI (in terms of age corrected W scores) and the acquired EEG frequencies in both groups.

## Results

### MRI

Table 1 shows the sociodemographic and neuropsychological characteristics of MCI subgroups defined by the tertile values of alpha3/alpha2. The ANOVA analysis showed that there were no statistically significant differences between groups which paired for age, sex, white matter hyperintensities (WMHs) burden, education, or global cognitive level. In addition, age, sex, education, global cognitive level (MMSE score), and WMHs were introduced as covariates in the subsequent analysis to avoid confounding factors. Alpha3/alpha2 ratio levels were significant using Games-Howell post hoc comparisons (p < 0.0001).

#### Pattern of cortical thickness between groups

**Fig. 1:**
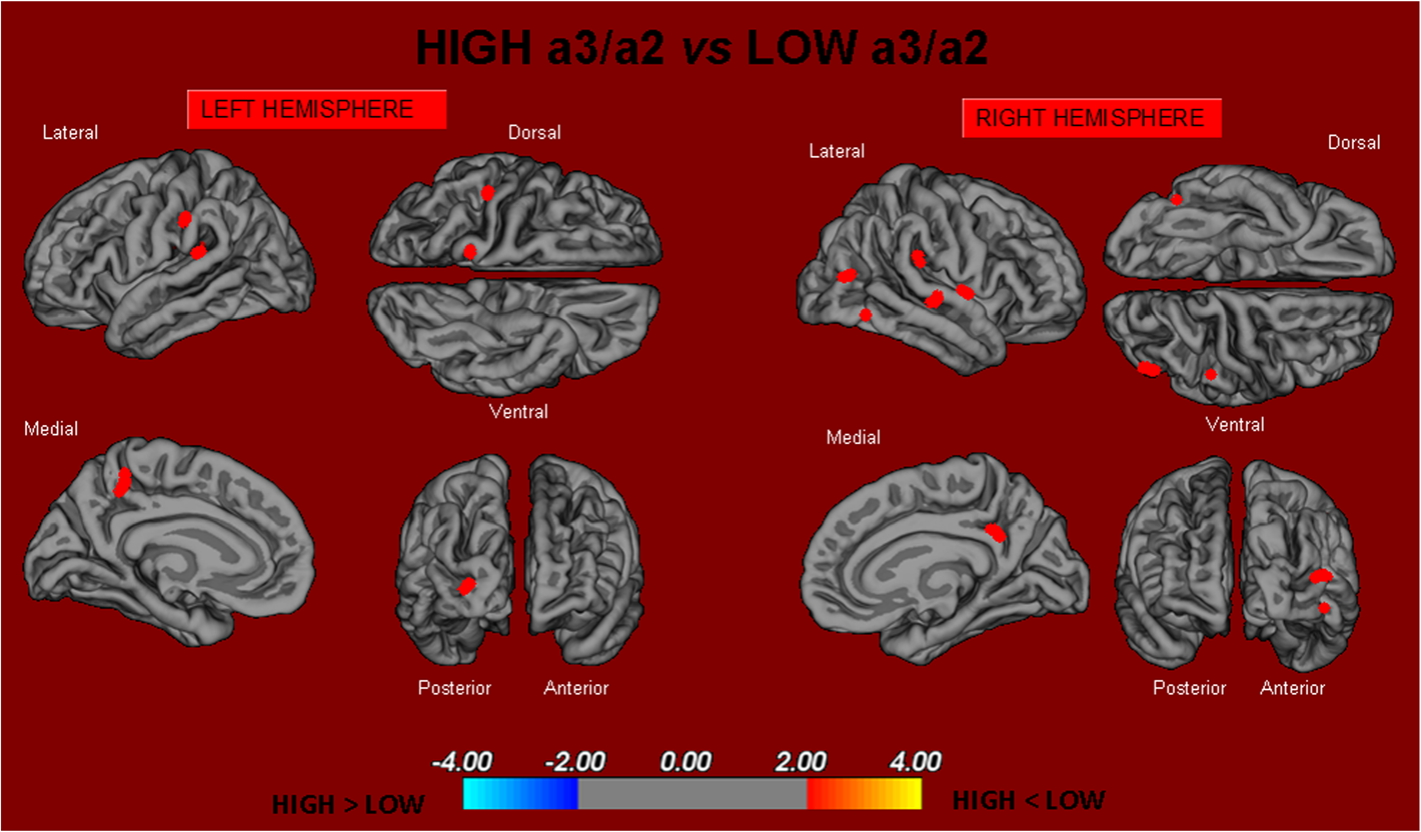
In red are represented the brain regions with higher regional cortical thickness in MCI patients with high a3/a2 ratio as compared to MCI patients with low a3/a2 ratio (p < 0.01 uncorrected). The color-coding for *p* values is on a logarithmic scale. Results are presented on the pial cortical surface of the brain: dark gray regions represent sulci and light gray regions represent gyri. *MCI* mild cognitive impairment
ii. High vs Middle: The same group showed a similar but less wide pattern of cortical thinning when compared to the middle a3/a2 group: the regions of atrophy were located in the left supramarginal gyrus, left precuneus, and postcentral cortex. The total CGM reduction in the high a3/a2 group compared to the middle a3/a2 group was 160 mm^2^ (Fig. 2). When the high group was compared to the low group, the total extent of cortical thinning (471 mm^2^) was 34 % larger than in the comparison of the high group to the middle group (160 mm^2^). No regions of major cortical atrophy were found in groups with middle or low a3/a2 power ratio when compared to the high a3/a2 group. No significant cortical thickness differences were found between middle and low a3/a2 groups.

i. High vs Low: When compared to subjects with low a3/a2 ratios, patients with high a3/a2 ratio show thinning of the bilateral superior temporal, supramarginal, and precuneus cortices, in the right inferior parietal cortex and insular cortex. The total cortical gray matter (CGM) reduction in the high a3/a2 group compared to the low a3/a2 group was 471 mm^2^ (Fig. 1).

**Fig. 2:**
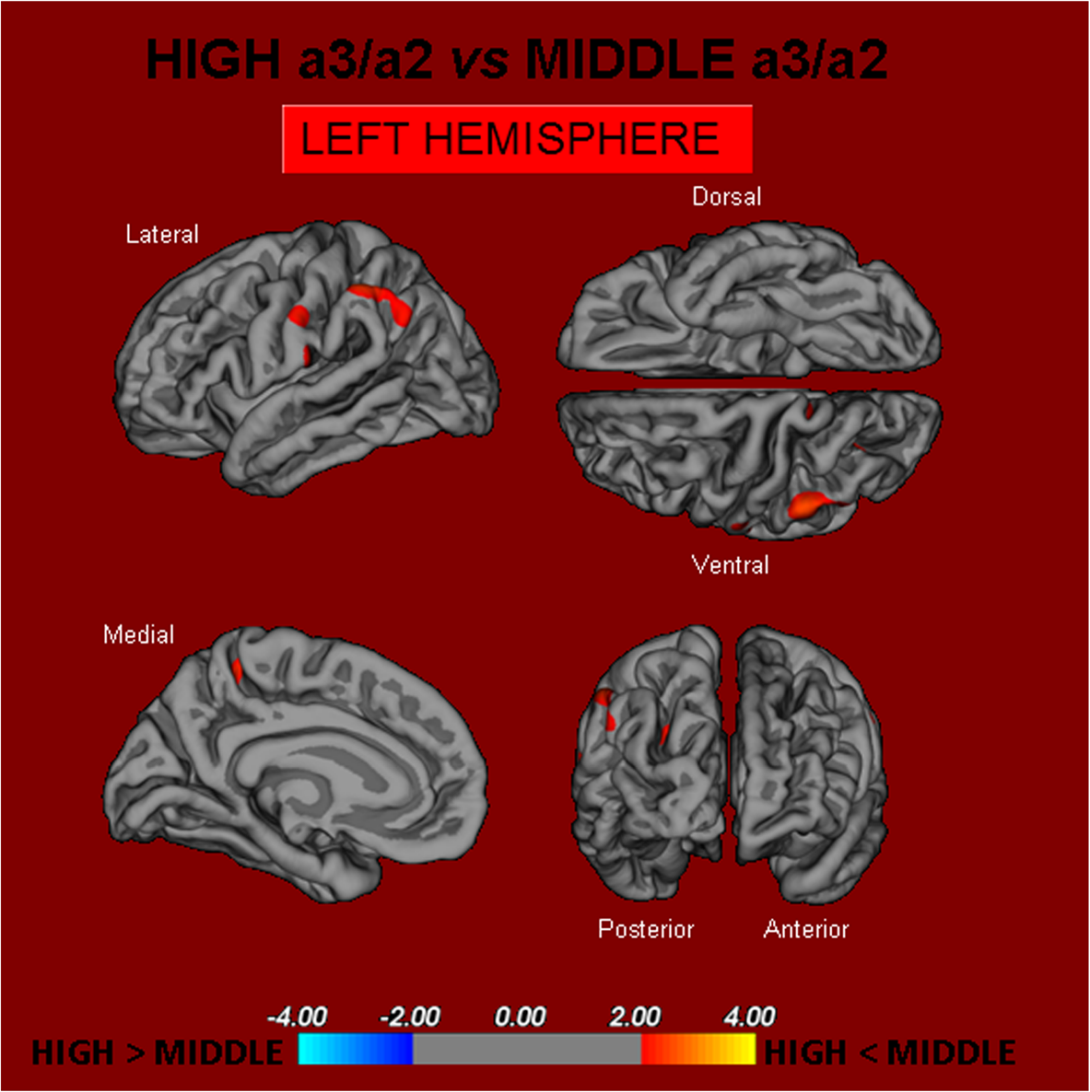
In red are represented the brain regions with higher regional cortical thickness in MCI patients with high a3/a2 ratio as compared to MCI patients with middle a3/a2 ratio (p < 0.01 uncorrected). The color-coding for *p* values is on a logarithmic scale. Results are presented on the pial cortical surface of the brain: dark gray regions represent sulci and light gray regions represent gyri. *MCI* mild cognitive impairment

#### Correlations between neuropsychological memory tests and cortical thickness in the high a3/a2 group and other groups

1. Babcock Test: In the high alpha3/alpha2 group, a significant positive correlation was found between logical memory performance in the Babcock test and thickness values in the left caudal middle frontal cortex (cluster size = 36 mm^2^; stereotaxic coordinate x, y, z = −34 22 47; r = 0.80; p = 0.0001) and left inferior temporal cortex (15 mm^2^; −54 -28 -26; r =072; p =0001), and right rostral middle frontal cortex (28 mm^2^; 23 56–13; r = 0.74; p = 0.00) (Fig. 3). No significant correlation was found within the same regions nor in the other groups nor in the whole sample. 2) AVLT immediate recall: In the high alpha3/alpha2 group, memory performance was significantly correlated with the cortical thickness values in the precuneus bilaterally (left 47 mm^2^; −21 -61 ; r = 0.78 < 0.000, right 58 m^2^; 20–60 25; r = 0.72 = 0.007, left fusiform cortex (40 mm^2^; −41 -25 -21; r = 0.76; p = 0005), inferior infertile parietal 43 mm^2^ -46 -60 11; r = 0.74; p = 0.0001), inferior temporal cortex (35 mm^2^; 21; r = 0.71; p = 0.0008), and the right bank of the superior temporal sulcus (44 m8 9; r = < 0.000). Memory performance was correlated in the middle group with both the right precuneus cortex (r = 0 = 0.03), and banks of the superior temporal sulcus (r = 0..02). No other associations were found in either the low group or in the entire sample.

**Fig. 3:**
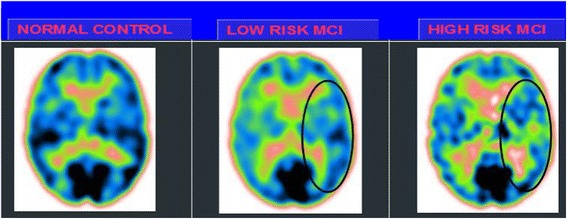
SPECT visual rating. The output shows a SPECT visual inspection of glucose uptake metabolism: the *black square* denotes an area of mild-to-moderate temporo-parietal hypometabolism in one of the 14 low risk MCI patients and in one of the 13 high risk MCI patients respective to one of the 17 enrolled controls. *SPECT* single-photon emission computed tomography, *MCI* mild cognitive impairment
2. AVLT delayed recall: In the high alpha3/alpha2 group, memory function correlated significantly with cortical thickness in the inferior parietal cortex bilaterally (left: 95 mm^2^; −44 -58 12; r = 0.86; p < 0.0000; right: 49 mm^2^; 50–50 9; r = 0.74; p = 0.0005), left pericalcarine cortex (54 mm^2^; −7 -8 11; r = 0.76; p < 0.0000), the banks of the superior temporal sulcus (31 mm^2^; −51 -41 -5; r = 0.81; p = 0.0002), and in the right superior temporal cortex (22 mm^2^; 56–34 13; r = 0.73; p = 0.001) . No significant correlation was found within the same regions in the other groups or in the whole sample.

### SPECT

Twenty seven MCI patients were enrolled in the present study and classified as at high risk (when the a3/a2 EEG rhythm median was above 1.17; N = 13) or at low risk (when the a3/a2 EEG rhythm median was under 1.17; N = 14) to develop AD. The two groups were similar in age (p = 0.56), education in years (p = 0.87), gender (p = 0.17), ApoE genotype (p = 0.15), MMSE scores (p = 0.31), and white matter lesions load (p = 0.88; Table 3). Figure 3 shows the visual rating scale of the SPECT scans representative of normal control, MCI with low risk to convert to AD, and MCI with high risk to convert to AD. ANOVA results show that the selected cut-off was effective in detecting two different groups: patients with high risk to develop AD show significantly higher alpha3/alpha2 power ratio than patients with low risk (p = 0.0001). Moreover, a control analysis was performed on the single frequencies. The results show that the increase of alpha3/alpha2 frequency power ratio was due to both increase of alpha3 (p = 0.001) and decrease of alpha2 (p = 0.0001) and not to the modification of a single frequency. This control analysis was performed because the change of only one frequency could be due to chance, but this was not the case.

**Table 3:** Demographic and cognitive characteristics of the SPECT subjects sample, disaggregated for increased levels of alpha3/alpha2

Demographic and clinical features	At low-risk	At high-risk	p
	MCI	MCI	
Number	14	13	
Age (years) [Range]	69.1 ± 7.6 [57÷83]	70.6 ± 5.5 [62÷78]	0.555
Gender (females)	6 (43 %)	9 (69 %)	0.168
Education (years) [Range]	8.2 ± 4.3 [4÷18]	7.9 ± 4.5 [3÷18]	0.865
MMSE score [Range]	27.9 ± 1.6 [25÷30]	27.2 ± 1.9 [24÷29]	0.309
ApoE ε4 genotype (carriers)	2 (29 %)	5 (39 %)	0.152
Left hippocampal volume (mm^3^) [Range]	2,606 ± 353 [1,923÷3,017]	2,073 ± 412 [1,234÷2,641]	0.001
Right hippocampal volume (mm^3^) [Range]	2,581 ± 473 [1,549÷3,150]	2,296 ± 501 [1,589÷3,086]	141
Wahlund total score [Range]	3.58 ± 3.29 [0.0÷10.0]	3.78 ± 2.63 [0.0÷7.0]	0.886

Numbers denote mean ± standard deviation, number and [range]. p denotes significance on ANOVA

*SPECT* single-photon emission computed tomography, *MCI* mild cognitive impairment, *MMSE* Mini-Mental State Examination, *ApoE* apolipoprotein E

Of note, no differences were found for beta 1, beta 2, gamma, theta EEG power, or theta/gamma frequency power ratio (all p > 0.11). Although the mean perfusion in all the selected ROIs was similar between groups (all p > 0.38), in the group with high alpha3/alpha2 frequency ratio, there is a constant trend to a lower perfusion. Moreover, left hippocampal volumes were lower for AD-high risk patients compared to low-risk patients (p = 0.001).

In patients at low risk to develop AD, a significant Pearson’s r negative correlation was found between perfusion in the hippocampal complex ROI and theta rhythm (r = −0.544, p = 0.044).

In patients at high risk to develop AD, other correlations were found. In contrast to patients at low risk, we found a positive correlation between perfusion in the hippocampal complex ROI and theta rhythm (r = 0.729, p = 0.005) in high risk patients while temporal ROI correlated positively with theta/gamma ratio rhythms (r = 0.736, p = 0.004) in this group. No other significant correlations were found in either group between perfusion ROIs and other EEG rhythms or hippocampal volumes. Moreover, no significant correlations were found between hippocampal complex ROI and theta rhythm pooling in low and high-risk patients together (r = 0.086, p = 0.671).

#### Correlations between neuropsychological memory tests and regional brain perfusion in the high a3/a2 group and other groups

1. Babcock Test: In the high alpha3/alpha2 group, a significant positive correlation was found between logical memory performance in the Babcock test and lower perfusion values in precuneus bilaterally (0.63 p = 0.03;) and superior temporal sulcus bilaterally (r = 0.74, p = 0.005). Moreover, a positive correlation was also found with hippocampal atrophy (r = 0.75, p = 0.001).
2. AVLT immediate recall: In the high alpha3/alpha2 group, memory performance was significantly correlated with lower perfusion values in the caudal bank of the right inferior temporal sulcus and middle frontal gyrus ( r = 0.75, p = 0.003).
3. AVLT delayed recall: In the high alpha3/alpha2 group, memory function correlated significantly with lower perfusion values in the inferior parietal lobule, particularly in the supramarginal gyrus (r = 0.09, p = 0.05).

#### Follow-up results

**Fig. 4:**
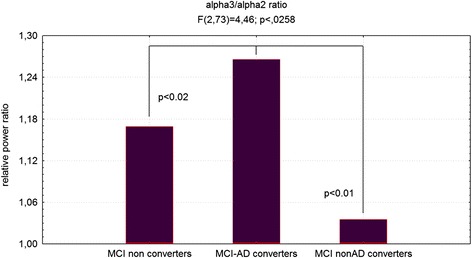
ANOVA results of theta/gamma and alpha3/alpha2 relative power ratio. In the graph post-hoc results are shown (see text for details). *ANOVA* analysis of variance

Figure 4 showed ANOVA significant statistical difference of theta/gamma and alpha3/alpha2 relative power ratio (F2,73 = 3.70; p < 0.03 and F2,73 = 4.46; p < 0.02, respectively). Duncan’s post-hoc test showed a significant increase (p < 0.01) of theta/gamma in both the MCI converters group with respect to non-converters as well as a significant increase of alpha3/alpha2 ratio in the MCI-AD converters with respect to the other groups (p < 0.02). In order to strengthen the results, the analysis was repeated also covarying the hippocampal volume and neuropsychological tests (NPS) scores. Significant results were confirmed for both theta/gamma (F2,73 = 2.49; p < 0.05) and alpha3/alpha2 ratio (F2,73 = 3.15; p < 0.04).

**Fig. 5:**
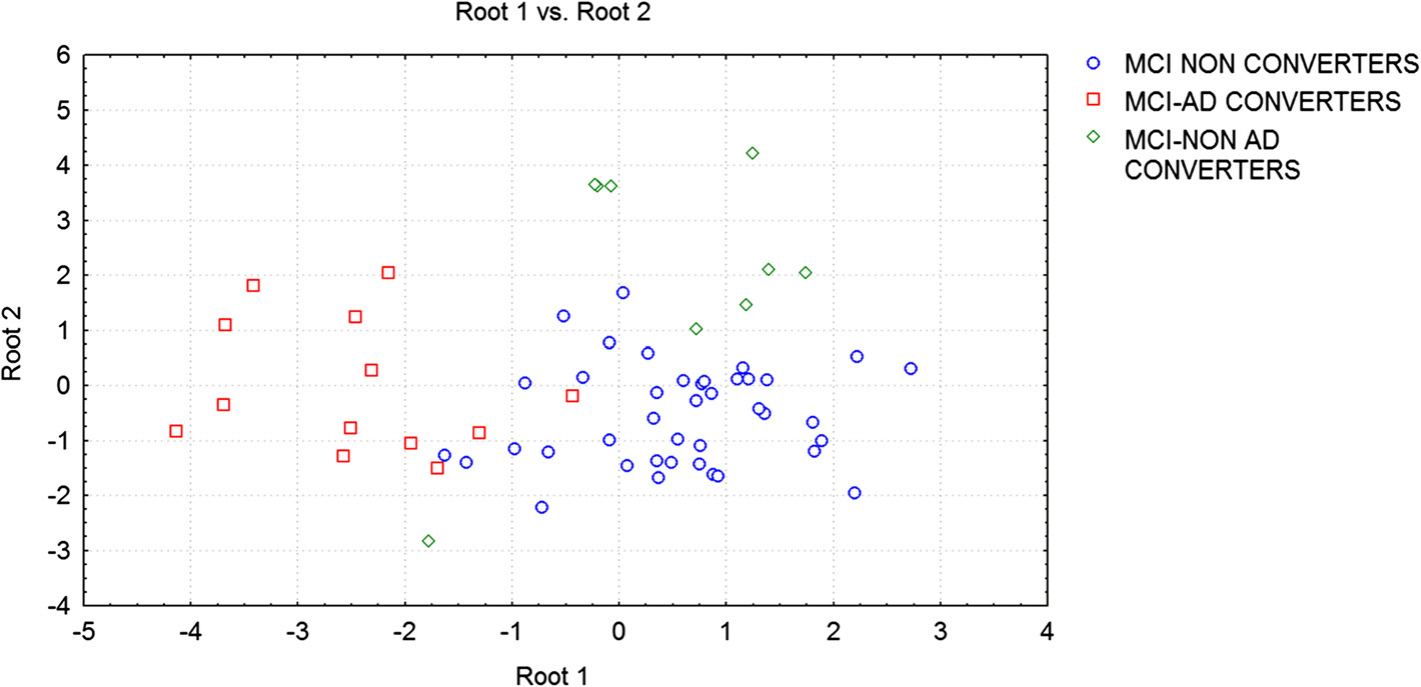
Discriminant factor analysis results: root1 vs root2 (see text for details)

The results of discriminant factor analysis show a statistically significant result for the model (F30,185 = 11.16; p < 0.00001; Wilks Lambda 0.04). A correct classification of groups was performed in the 85 % of cases in the first group, in the 94 % in the second group, and in the 86 % in the third group. The mean correct percentage of correct classification was 88.3 %. Based on the model structure, the variables accepted, ordered for statistical significance, were: theta/gamma frequency ratio (p < 0.0003) and alpha3/alpha2 frequency ratio (p < 0.03). EEG markers, together with duration of disease, were the most powerful variables in discriminating groups. The canonical analysis shows that the variables accepted were arranged in two discriminant functions (or roots). The two roots were all statistically significant (root1, p < 0.0001, root2, p < 0.001). Figure 5 shows a scatterplot of canonical scores (root1 vs. root2). The factor loadings of the variables on each discriminant function, as addressed by the factor structure matrix, shows that in root1 the theta/gamma relative power ratio has positive (0.5), and alpha3/alpha2 relative power ratio has negative, correlations (−0.7) whereas in root2 the theta/gamma relative power ratio has negative, (−0.6) and alpha3/alpha2 relative power ratio has positive (0.8), correlations with the discriminant function. As a consequence, an increase of theta/gamma relative power ratio better identifies the third group, and the increase of alpha3/alpha2 relative power ratio better identifies especially the second group.

## Discussion

### Study limitations

It needs to be remarked that our results about the EEG biomarker alpha3/alpha2 relative power ratio could have been emphasized given that a single class of dementia is considered. Of note, a recent study has demonstrated that the predictive value as well as the clinical application of the EEG biomarkers has to be proved in studies encompassing different forms of dementia [24]. Moreover, it should be pointed out that in our electrode array FP3, FP4, and inferotemporal zygomatic electrodes, which could provide information on hippocampal activity, were not present. This aspect could have limited the data collected in the study. However, the main scope of the study was the recording and analysis of cortical activity, fully mirrored by the electrode array utilized in the present study.

### Association between EEG markers and gray matter changes

In the present study, the relationship between an EEG marker (the alpha3/alpha2 power ratio) and the cortical thickness in subjects with MCI was investigated. The alpha3/alpha2 power ratio was selected because previous work has demonstrated that MCI subjects with a higher alpha3/alpha2 ratio are at major risk of developing AD [25–29]. Our results show that the MCI group with the higher alpha3/alpha2 ratio has a greater global cortical atrophy than the other subgroups, thus confirming a large body of literature [25–31]. Furthermore, the greater atrophy is significant in two specific brain areas: the precuneus and the supramarginal gyrus (a brain area belonging to the inferior parietal lobule), on both the left and right hemispheres. These results were largely expected considering previous studies. Indeed, structural and functional abnormalities of the precuneus were observed in MCI [32] as well as in AD [33] so that atrophy of the precuneus has been considered a pathognomonic marker of early AD.

### Association between EEG markers and perfusional changes

These results confirm previous studies that have shown that patients at high risk of developing AD have reduced SPECT perfusion in the temporo-parietal cortex and inferior parietal lobule [21]. The present study shows a correlation between cerebral perfusion and theta rhythm. However, the correlation emerges only when considering the different groups individually using the alpha3/alpha2 frequency power ratio. This is confirmed by the finding that when the groups are merged, no correlation could be found. This is the main aspect of the study and the peculiar novelty of the results. The patients at lower risk to develop AD, who have a constant trend towards a higher brain regional blood perfusion, maintain low levels of hippocampal theta power while in patients at higher risk, with a basically lower cerebral blood perfusion, theta rhythm tends to be higher in the hippocampus. Theta rhythms are usually not appreciated in normal awake EEGs. However, a theta power increase is observed over the frontal and temporal areas during learning and memory tasks [34]. The theta rhythms that are recorded during these tasks are thought to be produced by the activation of the septal-hippocampal system. The hippocampus has a cholinergic innervation originating from the basal forebrain, the medial septum, and the vertical limb of the diagonal band of Broca. Populations of GABAergic and glutamatergic neurons have also been described in several basal forebrain structures. The synchronized depolarization of hippocampal neurons produces field potentials that have a main frequency of 3–12 Hz and are usually known as hippocampal theta rhythm [34]. A cholinergic-glutamatergic hypothesis of AD, in which cholinergic-glutamatergic deficits may explain most symptoms, has been advanced. Neuronal injury/loss may include an excitotoxic component that possibly contributes to the early cholinergic deficit. This excitotoxic component may occur, at least in part, at the septal level where the somas of cholinergic neurons are found. This insult may modify septal networks and contribute to the abnormal information processing observed in AD brain, including its hyperexcitability states.

### Neurophysiological implications

Recent studies have demonstrated that during the successful encoding of new items, there is a desynchronization in the temporo-parietal memory-related networks, whereas synchronization prevents a successful semantic encoding [35]. The deleterious role of synchronization has been recently demonstrated by an interesting study of the intriguing relationship between functional and structural degeneration in AD. The authors detected some hub regions (heteromodal associative regions) with selective vulnerability to AD pathology due to the damage of inhibitory interneurons providing a loss of inhibition at the cellular level. According to the authors, the disinhibition provokes an increasing amount of neural activity at the network level, giving a hypersynchronization of brain areas as a final result. Of note, this overactivity is excitotoxic and determines cellular apoptosis and brain atrophy [36]. Also, Palop and Mucke emphasize the role of inhibitory interneuron dysfunction leading to hypersynchronization [36]. Our results are in line with these previous influential studies. A possible integrative view of all the results could be as follows: 1) the higher neuronal activity in the hub regions starts from a dysfunction of cellular inhibition; 2) the consequent disinhibition drives the neural network to an oversynchronization; 3) this oversynchronization is unique to the hub regions with higher amyloid burden; 4) these overactivated regions are prone to degeneration and atrophy; and 5) a possible neurophysiological sign of this oversynchronization is the increase of the alpha3/alpha2 power ratio we have found in typical hub regions [37–40]. It is of great interest that there is an overlap between the brain regions associated with the increase of the EEG alpha3/alpha2 power ratio (hypersynchronization of upper alpha) in our study and the regions associated with higher amyloid burden related to memory processes [41]. Moreover, in the present study, it is interesting that the atrophy of the precuneus is coupled with atrophy in the supramarginal gyrus and, to a lesser extent, the inferior parietal cortex, insula, and superior temporal gyrus. This atrophy pattern is clearly expressed in the group of MCI subjects with a higher alpha3/alpha2 power ratio. This finding fits well with the results of a recent study investigating the functional connectivity of human precuneus by resting state fMRI [41]. The authors found that there was a preferential pathway of connectivity of the dorsal precuneus with the supramarginal gyrus, parietal cortex, superior temporal gyrus, and insula. Consequently, the atrophy we detected in the MCI group with higher alpha3/alpha2 power ratio could be hypothesized as the loss of GM in an entire anatomo-functional network as opposed to atrophy of isolated brain areas. Of note, it is widely accepted that AD is the result of a cortical network impairment more than the atrophy of single cortical areas [41].

### Memory performance

In order to exclude a random relationship between EEG markers and cortical atrophy, the correlation between brain areas and the performance on memory tests was investigated in all MCI subgroups. The memory tests were selected because of their well-known greater impairment in MCI subjects who will convert to AD [1]. Our results show no significant memory differences among the groups. This could be a paradoxical outcome. However, it could not be considered a surprising result, taking into account the globally mild and early impairment of the whole group of subjects. In other words, when considering strictly the memory performance, the groups are not different. This is probably due to the early and mild cognitive impairment. However, despite no significant differences in the memory test scores, when focusing on the relationship between memory performance and a reliable structural marker, such as the cortical thickness, the MCI group with the higher alpha3/alpha2 power ratio has shown a (negative) correlation between memory test performance and the cortical thickness, as expected in patients with probable prodromal AD. This result confirms the peculiar nature of this MCI group, showing a clear specificity concerning both the cortical atrophy and the correlated memory performance. Moreover, no other socio-demographical or structural differences were observed in the MCI groups that could explain the correlation analysis results. The cortical areas associated with cortical thinning and those correlated with memory test performances are only partly overlapping. This could be due to the particular nature of the memory domain, underpinning a large number of brain areas. On the other hand, MCI subjects more susceptible to convert to AD could also show impairment in other cognitive domains, such as visuospatial attention or execution and preparation of spatially guided behavior [41]. Of note, the cortical network encompassing precuneus and inferior parietal cortex is deeply involved in visuospatial abilities and left hippocampal atrophy [41]. As a speculative interpretation, we could hypothesize that the memory deficits are due to an impaired network underlying the semantic coding of the spatial features of the episodic memory traces. In this view, the atrophy of a specific brain network (more than global volume measures) is more reliable in detecting MCI subjects with prodromal AD. However, the discussion of memory-related brain networks is beyond the scope of the present study. Only a weak negative correlation was found in the middle alpha3/alpha2 EEG power ratio group, suggesting a possible degenerative nature of the memory impairment in this group. No significant associations were found in the low alpha3/alpha2 power ratio group and the whole sample. Taken together, these results strengthen the position of the higher alpha3/alpha2 ratio MCI group as being at major risk of developing Alzheimer’s disease.

### Diagnostic implications

After three years of follow-up, three subgroups were characterized as converters to Alzheimer’s disease (AD, N = 18), converters to non-AD dementia (N = 14) and non-MCI to AD converters (N = 42). Increased alpha3/alpha2 ratio was only associated with conversion to AD. EEG markers allow a mean correct percentage of correct classification up to 88.3 %. Future prospective studies are needed to evaluate the sensitivity and specificity of these measures for predicting an AD outcome.

### Implications at the system level

Klimesch and coworkers have convincingly demonstrated that the upper alpha band (10–13 Hz) specifically reflects encoding memory processes [42]. Recent EEG and magnetoencephalography (MEG) studies have confirmed that a correct functioning of memory, both in encoding and in retrieval, requires a high alpha rhythm desynchronization (or power decrease) [42]. From a neurophysiological point of view, the synchronization (or power increase) of EEG alpha power has been associated with the inhibition timing hypothesis [43] and with poor information transmission, according to the entropy theory [44]. Applying this concept to the neural networks, it has been demonstrated that the degree of information that is encoded in neural assemblies increases as a function of desynchronization and decreases as a function of synchronized firing patterns This hypothesis has been confirmed in clinical studies in patients with memory deficits [45] as well as during states where there is little cognitive processing (e.g., epileptic seizures or slow wave sleep) [46]. Concerning cognitive impairment due to AD, the typical synaptic losses could prevent the physiological flexibility of brain neural assemblies, impeding the desynchronizing downstream modulation of the brain activity. Therefore, it could be hypothesized that the disruption of the cortical network due to degenerative disease, inducing cortical atrophy, could determine an oversynchronization of the brain oscillatory activity. The synchronization state of the high alpha power could prevent the creation of a semantic sensory code and, consequently, of the episodic memory trace. Of note, according to the new diagnostic criteria for AD, the measurement of sensitivity to semantic cueing can successfully differentiate patients with AD from healthy controls, even when patients are equated to controls on MMSE scores or when disease severity is very mild. Our results are in line with this hypothesis, suggesting that an increase in the power of high alpha brain oscillations reflects a block of information processes. However, the present study goes one step further, linking the increase of high alpha synchronization to the atrophy of a specific brain network, correlated with impairment in memory performances and decreased cerebral blood flow activity.

### Preliminary remarks

There are some limitations and caveats in the present study: (1) further studies are needed to confirm our results on larger samples and to apply appropriate multiple comparison corrections; (2) the pattern of cortical thickness should be investigated on the remaining EEG frequency measures; (3) the conservative p < 0.001 used here is not necessarily sufficient given the number of comparisons. Given the explorative nature of the study it is plausible to use a permissive approach in order to avoid the rejection of possibly interesting results. It remains clear that further studies with a less permissive statistical approach are mandatory to confirm our results. Of note, the reliability of the results is supported by: (1) the rigorous selection criteria of the subjects; (2) the high statistical threshold (p < 0.001) considered; (3) the large size of pixel accounted for the analysis (30 mm2); and (4) finally, the statistical control analysis represented by the correlation of cortical thickness with memory tests.

## Conclusions

A high EEG upper/low alpha power ratio was associated with cortical thinning and lower perfusion in the temporo-parietal lobe. Moreover, atrophy and lower perfusion rate were both significantly correlated with memory impairment in MCI subjects. The increase in the upper/low alpha frequency power ratio could be useful for identifying individuals at risk for progression to AD dementia and may be of value in the clinical context.

## Abbreviations

99mTc-ECD: Technetium-99 m ethyl cysteinate dimer
AD: Alzheimer’s disease
ANOVA: Analysis of variance
ApoE: Apolipoprotein E
AVLT: Auditory verbal learning test
CGM: Cortical Gray Matter
CSF: Cerebrospinal fluid
EEG: Electroencephalogram
EOG: Electrooculogram
FDG PET: Fluorodeoxyglucose positron emission tomography
FFT: Fast Fourier Transform
FTD: Fronto-temporal dementia
HIS: Hachinski Ischemic Scale
HV: Hippocampal volume
IADL: BADL, Instrumental and Basic Activities of Daily Living
IAF: Individual alpha frequency
LBD: Lewy body dementia
MCI: Mild cognitive impairment
MEG: Magnetoencephalography
MRI: Magnetic resonance imaging
ROI: Regions of interest
SPECT: Single-photon emission computed tomography
SPM: Statistical parametric mapping
TF: Transition frequency
TOMC: Translational outpatient memory clinic
VD: Vascular dementia
WMHs: White matter hyperintensities
